# Acute hypoxia elicits prefrontal oxygenation asymmetry in young adults

**DOI:** 10.1117/1.NPh.10.4.045002

**Published:** 2023-10-05

**Authors:** Kojiro Ide

**Affiliations:** Hokusho University, School of Lifelong Sport, Northern Region Lifelong Sports Research Center, Hokkaido, Japan

**Keywords:** frontal cerebral oxygenation asymmetry, isocapnic hypoxia, cerebral hemodynamic, heart rate, prefrontal cortex

## Abstract

**Significance:**

Cerebrovascular reactivity can be evaluated by prefrontal cortex (PFC) hemodynamic responses and oxygenation changes secondary to hypoxia using near-infrared spectroscopy (NIRS). However, whether there are hemispheric differences in these NIRS-determined PFC hemodynamic responses and oxygenation changes remains unknown.

**Aim:**

This study was performed to determine whether there are differences in the PFC hemodynamic responses and oxygenation changes secondary to hypoxia between the left and right frontal poles (FPL and FPR, respectively).

**Approach:**

Fifteen young men participated in the study. During conduction of an isocapnic hypoxia protocol with a 10-min hypoxic phase at partial pressure of end-tidal oxygen (${\mathrm{PET}}_{O_{2}}$) of 45 Torr, hemodynamic and oxygenation indices comprising oxygenated hemoglobin (oxy-Hb), deoxygenated Hb (deoxy-Hb), total Hb (total-Hb), and tissue oxygen saturation (${\mathrm{StO}}_{2}$) over FPL and FPR were measured by NIRS. The heart rate (HR) was evaluated by electrocardiography.

**Results:**

In response to hypoxia, the HR increased, oxy-Hb decreased, deoxy-Hb increased, total-Hb increased above baseline, and ${\mathrm{StO}}_{2}$ decreased. There was no difference in the change in total-Hb between FPL and FPR. However, there were greater changes in oxy-Hb, deoxy-Hb, and ${\mathrm{StO}}_{2}$ over FPL than over FPR, indicating that PFC oxygenation asymmetry occurs in response to hypoxia. Moreover, the change in total-Hb over FPL was associated with the increase in HR.

**Conclusions:**

NIRS-determined hemodynamic responses and oxygenation changes secondary to hypoxia might not simply reflect the direct effect of hypoxia on cerebral vessels. Although there is no hemispheric difference in the PFC hemodynamic responses to hypoxia as in total-Hb, PFC oxygenation asymmetry occurs in young adults.

## 1 Introduction

Cerebral vessels are sensitive to arterial blood gas alterations, such as hypoxia and/or hypercapnia. Some studies have shown that cerebrovascular reactivity (CVR) to hypercapnia is attenuated in older adults^1^ and in patients with endothelial disfunction,^2^ hypercholesterolemia,^3^ and dementia.^4^ Accordingly, it has been suggested that CVR is a potent physiological variable for cerebrovascular and cognitive health. The CVR to hypercapnia has been extensively evaluated using transcranial Doppler^5^ and functional magnetic resonance imaging (fMRI).^1^ Near-infrared spectroscopy (NIRS) has also been used to measure hemodynamic responses and oxygenation changes secondary to hypercapnia^6^ to evaluate CVR, and a similar trend has been observed in CVR determined by NIRS.^7^ NIRS is used to measure deoxygenated hemoglobin (deoxy-Hb) and oxygenated Hb (oxy-Hb) and to evaluate tissue oxygenation. Benefits of this method are its non-invasiveness, relatively low cost, and lack of need for high skill qualification.^8^^,^^9^ Despite these benefits, further research is needed to gain a better understanding of cerebral hemodynamic responses and oxygenation changes determined by NIRS.

NIRS has often been used to evaluate neurovascular coupling.^10^^–^^14^ A general finding is that the hemodynamics over the prefrontal cortex (PFC) during cognitive tasks is either right-lateralized^11^^–^^13^ or left-lateralized^14^ in young adults. Continuous performance tests are one example of cognitive tasks that elicit prefrontal laterality. Some studies have shown that increased hemodynamics during continuous performance tests using mental arithmetic is right-lateralized in young adults.^12^^,^^13^ Such increases in the hemodynamics over the PFC are accompanied by increases in the heart rate (HR). Moreover, the laterality of increased hemodynamics with right PFC dominance is associated with increased heart rate,^12^^,^^13^ changes in the parameters of sympathetic nerve activity [such as the ratio of low frequency to high frequency in HR variability (HRV)], and inverse changes in the parameters of parasympathetic nerve activity (such as high-frequency HRV).^12^ With respect to how the PFC is involved in HR and HRV changes, it has been postulated that the PFC affects changes in sympathetic and/or parasympathetic nerve activity to the heart via the central autonomic network (CAN).^15^

Hypoxia causes oxygen ($O_{2}$) to dissociate from Hb, resulting in increased deoxy-Hb and decreased oxy-Hb and tissue oxygenation. Cerebral vessels are sensitive to hypoxia, and hypoxia elicits vasodilatation and increases in cerebral blood flow (CBF). Such increases in CBF help mitigate decreases in cerebral tissue oxygenation associated with hypoxia. A study in which NIRS was used demonstrated these hemodynamic responses and oxygenation changes secondary to hypoxia.^16^ The HR also increases in response to hypoxia, and such increases in the HR in response to acute hypoxia are considered to be caused by sympathetic activation^17^ and/or vagal withdrawal.^18^ Accordingly, similar to the increased hemodynamics over the PFC and the HR responses to cognitive tasks, these physiological responses occur secondary to hypoxia as well. However, no previous studies have explored whether differences exist in the hemodynamic responses and oxygenation changes secondary to hypoxia between the right and left PFCs, or whether the changes in NIRS parameters are associated with HR responses.

With respect to differences in the hemodynamics and oxygenation between the right and left PFCs, Olopade et al.^19^ showed that there was no difference in NIRS-determined oxygenation between the right and left PFCs during sleep in either healthy adults or patients with obstructive sleep apnea. However, Zohdi et al.^20^ showed that there was a difference between the right and left PFCs in NIRS-determined oxygenation during the awake resting state, and such prefrontal oxygenation asymmetry is associated with the respiratory rate and pulse-respiration quotient.^21^ When considering whether this is also the case in hypoxia, only one study^22^ measured tissue oxygenation in both the right and left PFCs in response to hypoxia to compare two different NIRS devices (a continuous-wave NIRS device for the right PFC and a frequency-domain NIRS device for the left PFC). This study showed no difference in NIRS-determined oxygenation in response to hypoxia between the right and left PFCs.^22^

Although there is no clear indication regarding whether the hemodynamics and oxygenation in response to hypoxia as determined by NIRS differs between the right and left PFCs, one study that measured changes in regional CBF (rCBF) in response to hypoxia using fMRI indicated such a possibility. In that study, Critchley et al.^23^ demonstrated increases in rCBF in response to hypoxia over various regions, including the bilateral amygdala and the occipital and medial and dorsolateral PFCs; an increase in blood flow over the lateral frontal pole was seen only on the left side. Moreover, such regional differences in CBF may be associated with differences in neural activity in addition to the direct effect of hypoxia on cerebral vessels. Critchley et al.^23^ also investigated neural substrates of cardiorespiratory control during controlled breathing or spontaneous breathing under normoxia or hypoxia using fMRI and found that, after analyzing data across all conditions, changes in the left PFC as well as other areas (caudate/putamen and posterior insula) were associated with decreases in the HR.^23^ Accordingly, it can be hypothesized that there are differences between the right and left PFCs in the hemodynamic responses and oxygenation changes secondary to hypoxia and that the hemodynamic responses and oxygenation changes secondary to hypoxia are associated with the HR response.

Thus, this study was performed to investigate the differences in the hemodynamic responses and oxygenation changes secondary to hypoxia as determined by NIRS between the right and left PFCs and the relationship of these differences to changes in HR. One concern of this study was whether changes in skin blood flow may affect NIRS-determined hemodynamic responses to hypoxia because hypoxia enhances skin blood flow.^24^ Accordingly, NIRS was used with an optical probe that had a single emitter and two detectors with different emitter–detector intervals (4 and 35 mm) to simultaneously obtain NIRS signals from both the superficial and relatively deeper layers of the forehead. This study may provide an understanding of the clinical implications of the hemodynamic responses and oxygenation changes secondary to hypoxia as measured by NIRS for evaluation of CVR or cerebral oxygenation.

## 2 Subjects and Methods

### 2.1 Subjects

The study involved 15 young Asian men (age, 20.1±1.9 years; body weight, 64.6±6.2  kg; and height, 171.9±4.9  cm) (mean ± standard deviation). Recruitment advertisements for the study were placed on notice boards in the college, and the subjects contacted the investigator for a detailed explanation of the study. The study requirements were fully explained to all participants, and written informed consent was provided by each participant before participation in the study. The study protocol was approved by the ethics committee of Hokusho University and was conducted according to the Declaration of Helsinki. The participants were taking no medications, all were nonsmokers, and none had any history of cardiovascular, cerebrovascular, or respiratory disease.

### 2.2 Protocol

The subjects participated in a project to study the relationships among cerebrovascular reactivity, cognitive function, and aerobic capacity (Grant-in-Aid for Scientific Research (C) for 16K01729).

The subjects abstained from caffeine and alcohol for 12 h and vigorous exercise for 24 h prior to the study. They visited the laboratory at 9:00 AM and underwent measurements of body composition, arterial stiffness, cognitive function, hemodynamic response and oxygenation changes secondary to hypoxia, and aerobic capacity. The data regarding body composition, arterial stiffness, cognitive function, and aerobic capacity are not presented in this article.

### 2.3 Isocapnic Hypoxia Test

**Fig. 1:**
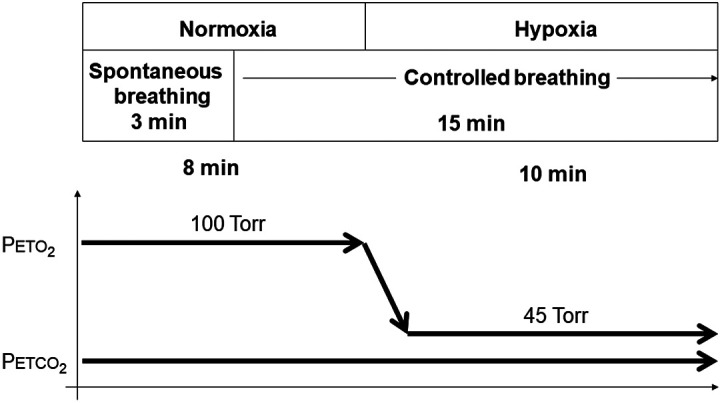
Experimental protocol of isocapnic hypoxia test. Abbreviations: ${\mathrm{PET}}_{{\mathrm{CO}}_{2}}$, partial pressure of end-tidal carbon dioxide; ${\mathrm{PET}}_{O_{2}}$, partial pressure of end-tidal oxygen.

Resting partial pressure of end-tidal carbon dioxide (${\mathrm{PET}}_{{\mathrm{CO}}_{2}}$) was measured while the participant sat quietly and comfortably for ∼10  min. Respired gas was sampled via a fine catheter held at the opening of one nostril by an adapted nasal $O_{2}$ therapy kit. The gas was sampled continuously and analyzed for carbon dioxide (${\mathrm{CO}}_{2}$) and $O_{2}$ (AE-310s; Minato Medical Science Co., Ltd., Osaka, Japan). An experimental protocol of isocapnic hypoxia was then performed (Fig. 1).

This protocol comprised 8 min of normoxia (baseline) and 10 min of hypoxia. While seated in a chair, the subjects breathed through a mouthpiece. The protocol was initiated with a 3-min period of the subject breathing normally through a mouthpiece with his nose occluded by a nose clip. The subject then started to control his respiratory rate to one breath every 4 s and maintained this rate until the end of the experiment. The time point at which to switch from inspiration to expiration and vice versa was indicated by an audio alarm. The composition of inspired and expired gas was continuously measured and monitored, and the composition of inspired gas was altered to establish isocapnic hypoxia. At baseline, the inspired gases were controlled to hold the partial pressure of end-tidal oxygen (${\mathrm{PET}}_{O_{2}}$) at ∼100  Torr and the ${\mathrm{PET}}_{{\mathrm{CO}}_{2}}$ as close as possible to the subject’s resting value that had been measured prior to the experiment. The fraction of inspired $O_{2}$ was then adjusted to decrease the ${\mathrm{PET}}_{O_{2}}$ to 45 Torr with an ∼10% fraction of inspired $O_{2}$, and the fraction of inspired ${\mathrm{CO}}_{2}$ was controlled to stabilize the ${\mathrm{PET}}_{{\mathrm{CO}}_{2}}$ at the baseline value with an ∼3% fraction of inspired ${\mathrm{CO}}_{2}$.

During the isocapnic hypoxia protocol, changes in oxy-Hb, deoxy-Hb, and total Hb (total-Hb) over the forehead were measured continuously using a wireless near-infrared spatially resolved spectroscope (Hb-13; ASTEM Co., Ltd., Kanagawa, Japan). The sampling frequency was 2 Hz. Probes of the emitter and detector were located to measure changes in Hb over the left frontal pole (FP1: FPL) and right frontal pole (FP2: FPR) using the international electroencephalographic (EEG) 10–20 system. Each optical probe comprised a single emitter and two detectors with different emitter–detector intervals (4 and 35 mm) to simultaneously obtain the NIRS signals from the superficial layer and relatively deeper layer of the head. Two wavelengths used for this device were 770 and 830 nm.^25^ A fundamental study for the development of this device^25^ and a study using the same device^26^ have been published previously. The probes were fixed on the forehead using a headband, and the whole head was then covered with a black cotton cap to prevent room light from reaching the probes. The extent to which the cap prevented light from reaching the probes was tested using the NIRS software.

Electrodes were placed over the chest to record a lead II electrocardiogram (ECG) for evaluation of HRV. Data were stored in a personal computer for later analysis. The analog signal of the ECG was conveyed through an analog-to-digital converter (PowerLab 8/35; ADInstruments, Dunedin, Otago, New Zealand) with a sampling rate of 1 kHz for offline analysis using software (LabChart; ADInstruments). The ECG data were analyzed offline using commercially available software (HRV Module for LabChart 8; ADInstruments). Data for analysis were extracted from the final 5 min of data of the 8-min baseline period and that of the 10-min hypoxic period. The following time domain parameters were computed: mean and standard deviation of the R–R interval in ms, mean of the HR in beats/min, square root of the mean squared differences in successive R–R intervals (RMSSD) in ms, and percentage of adjacent R–R intervals that varied by >50  ms (pRR50). The frequency domain parameters comprised the normalized very-low-frequency spectral power (VLF) in ${\mathrm{ms}}^{2}$ and %, low-frequency spectral power (LF) in ${\mathrm{ms}}^{2}$ and %, high-frequency spectral power (HF) in ${\mathrm{ms}}^{2}$ and %, and low-frequency to high-frequency ratio (LF/HF). The VLF component of HRV was obtained by integrating the power spectral density within the VLF frequency range (0.0033 to 0.04 Hz). The VLF component is influenced by a variety of physiological processes, including hormonal regulation, physical activity, and thermoregulation. The LF component was obtained by integrating the power spectral density within the LF frequency range (0.04 to 0.15 Hz). The LF component is influenced by both sympathetic and parasympathetic nervous system activity. The HF component was calculated by integrating the power spectral density within the HF frequency range (0.15 to 0.4 Hz). The HF component is predominantly influenced by parasympathetic nervous system activity and particularly by respiratory sinus arrhythmia. The percentage of VLF was calculated as (power in VLF)/(total power) * 100, the percentage of LF was calculated as (power in LF)/(total power) * 100, and the percentage of HF was calculated as (power in HF)/(total power) * 100; total power was the sum of power across all frequency bands.^27^ There were no ectopic beats, arrhythmic events, or noise effects that might have affected the HRV analysis.

### 2.4 Control of Inspired Gases and Breathing

End-tidal gases were controlled by feeding a gas mixture of air, nitrogen, and ${\mathrm{CO}}_{2}$. The gas mixture was made in a gas mixing chamber immediately before feeding using gas flow controllers (LMX2-J-A7; Front Co., Ltd., Tokyo, Japan) for each gas. The gas mixture was fed to one end of a two-way valve connected to the mouthpiece. One end of the two-way valve was connected to a gas mixing chamber of air, nitrogen, and ${\mathrm{CO}}_{2}$, and the other end was open for expired gas to escape into the room air. The inspired and expired gases were analyzed by a gas analyzer for the fractional concentrations of ${\mathrm{CO}}_{2}$ and $O_{2}$ and respiratory variables comprising the respiratory rate, total time for one respiratory cycle, and expiratory tidal volume (AE-310s; Minato Medical Science Co., Ltd.). Inspired and end-tidal gases were monitored continuously. The contents of the gas mixture were manually adjusted by hand using gas flow controllers (LMX2-J-A7; Front Co., Ltd.) instead of a computer control,^5^ depending on the level of end-tidal gases. Analog signals of ${\mathrm{CO}}_{2}$ and $O_{2}$ were imported into a personal computer for later analysis using an analog-to-digital converter (PowerLab 8/35; ADInstruments) and software (LabChart; ADInstruments).

### 2.5 Analysis

The data for the final 120 s of the baseline period before the onset of the hypoxic stimulus and for the entire hypoxic period were extracted for analysis. The data for changes in total-Hb, oxy-Hb, deoxy-Hb, ${\mathrm{PET}}_{O_{2}}$, and ${\mathrm{PET}}_{{\mathrm{CO}}_{2}}$ were averaged to provide one arithmetic mean value over each 60-s period. The data for the final 120 s of the baseline period before the onset of the hypoxic stimulus were averaged to provide a mean value to use as the baseline datum. These data of total-Hb, oxy-Hb, deoxy-Hb, and tissue $O_{2}$ saturation (${\mathrm{StO}}_{2}$) were normalized relative to that for the baseline by subtracting the baseline data from these data for the hypoxic period.

Data of the R–R interval to analyze the HR and HRV were extracted from the 5-min data immediately before the onset of the hypoxic stimulus and the final 5-min data for the hypoxic period. Changes in HRV from baseline to application of hypoxic stimuli were calculated by subtracting the data for the baseline period from the data for the hypoxic period.

### 2.6 Statistics

Changes in ${\mathrm{PET}}_{{\mathrm{CO}}_{2}}$ and ${\mathrm{PET}}_{O_{2}}$ were analyzed using repeated-measures analysis of variance (ANOVA) in conjunction with the Bonferroni *post hoc* test for multiple comparisons. The effects of hypoxia on changes in hemodynamics in the relatively deep layer of the forehead (time) and differences in hemodynamic responses between FPL and FPR (channel; Ch) were analyzed using two-way repeated-measures ANOVA (Ch × time) in conjunction with the Bonferroni *post hoc* test for multiple comparisons. The effects of hypoxia on changes in hemodynamics in the superficial layer over the forehead (time) and differences between FPL and FPR (Ch) were also analyzed using two-way repeated-measures ANOVA (Ch × time).

The normality of each HRV parameter, hemodynamics, and oxygenation was analyzed using the Shapiro–Wilk test. The effects of hypoxia on the parameters of HRV were analyzed using Student’s t-test or Wilcoxon’s signed rank test in accordance with the results of normality. To detect correlations between variables, Pearson’s product-moment correlation coefficient (R) analysis or Spearman’s rank order test was used in accordance with the results of normality. A P value of <0.05 was considered statistically significant, and the effect size was also provided. The effect size for ANOVA was provided by partial $\eta^{2}$ using a statistical software package. Student’s t-test, product-moment correlation coefficient (R) analysis, repeated-measures ANOVA, and two-way repeated-measures ANOVA were performed with IBM SPSS Statistics, version 24.0 (IBM Corp., Armonk, New York). G*Power was used to perform the statistical power analysis after collecting the data.^28^^,^^29^

## 3 Results

### 3.1 Changes in End-Tidal Gases, Changes in HR, and NIRS-Determined Hemodynamics Responses and Oxygenation Changes Secondary to Isocapnic Hypoxia

**Fig. 2:**
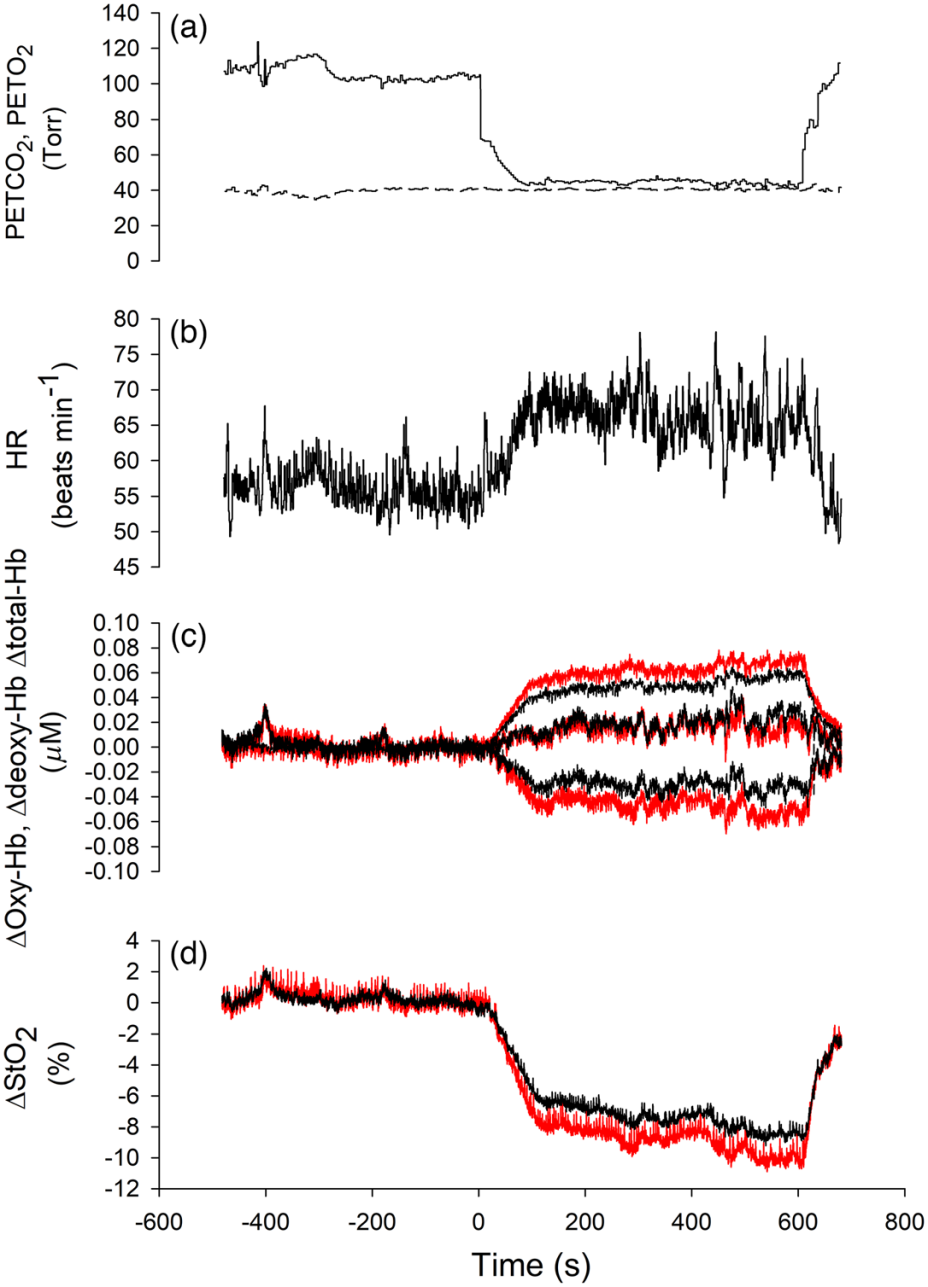
Typical example of time traces of changes in (a) ${\mathrm{PET}}_{O_{2}}$, ${\mathrm{PET}}_{{\mathrm{CO}}_{2}}$, and (b) HR, as well as normalized changes in (c) deoxy-Hb, total-Hb, oxy-Hb, and (d) ${\mathrm{StO}}_{2}$ in the relatively deeper layer over the frontal poles in relation to time after baseline. (a) Solid line: ${\mathrm{PET}}_{O_{2}}$, dashed line: ${\mathrm{PET}}_{{\mathrm{CO}}_{2}}$. (c), (d) Red solid lines: oxy-Hb, deoxy-Hb, total-Hb, and ${\mathrm{StO}}_{2}$ over FPL; black solid lines: oxy-Hb, deoxy-Hb, total-Hb, and ${\mathrm{StO}}_{2}$ over FPR. Abbreviations: ${\mathrm{PET}}_{{\mathrm{CO}}_{2}}$, partial pressure of end-tidal carbon dioxide (Torr); ${\mathrm{PET}}_{O_{2}}$, partial pressure of end-tidal oxygen (Torr); HR, heart rate (beats/min); oxy-Hb, oxygenated hemoglobin; deoxy-Hb, deoxygenated hemoglobin; total-Hb, total hemoglobin; ${\mathrm{StO}}_{2}$, tissue oxygen saturation; FPL, left frontal pole; FPR, right frontal pole.

**Fig. 3:**
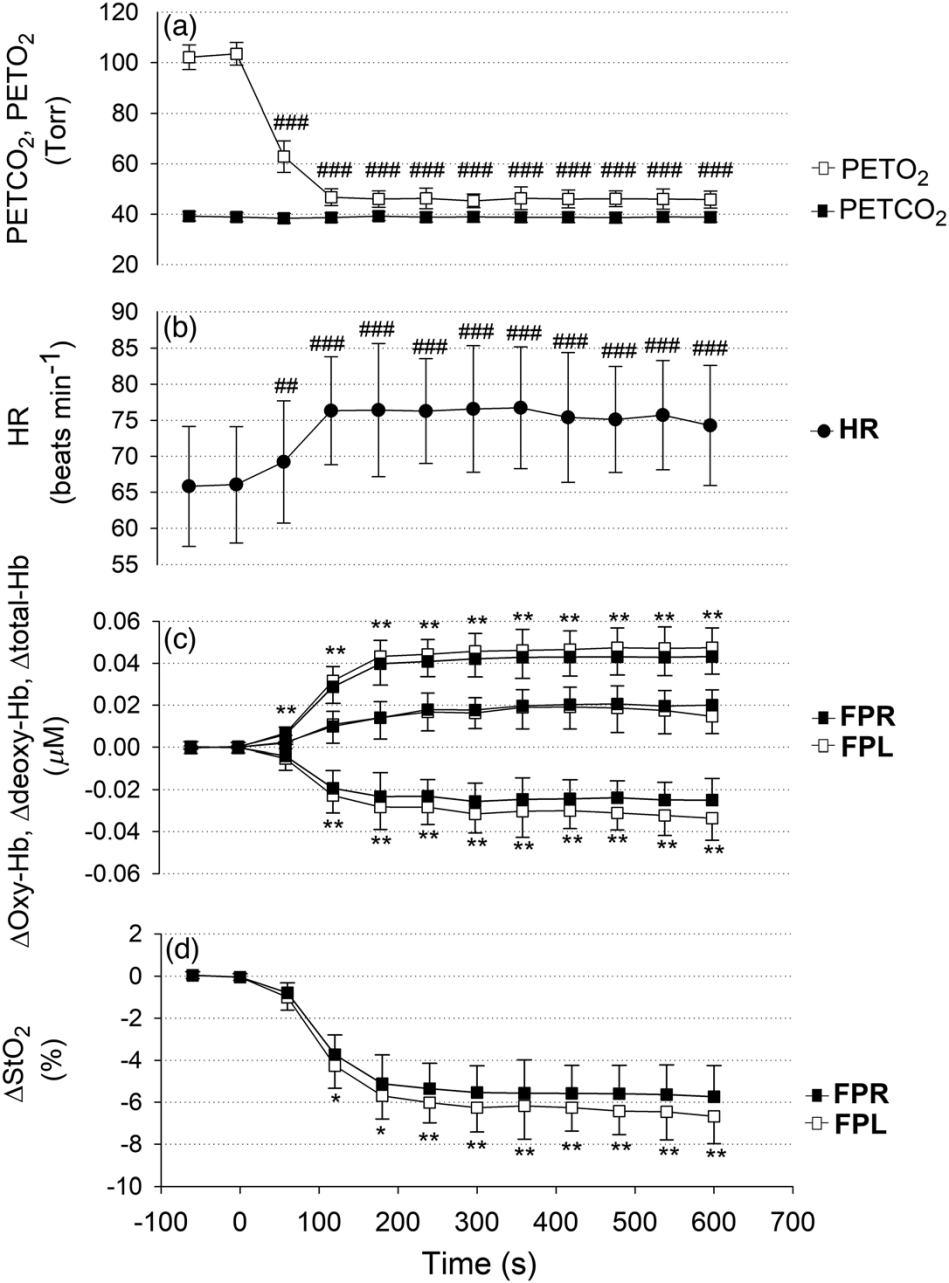
Average time traces of changes in (a) ${\mathrm{PET}}_{O_{2}}$ and ${\mathrm{PET}}_{{\mathrm{CO}}_{2}}$, (b) HR and normalized changes in (c) deoxy-Hb, total-Hb, oxy-Hb, and (d) ${\mathrm{StO}}_{2}$ in the relatively deeper layer over the frontal poles in relation to time after baseline. Each symbol and error bar represents the 60-s mean and SD among n=15 subjects. Open squares: ${\mathrm{PET}}_{O_{2}}$; solid squares: ${\mathrm{PET}}_{{\mathrm{CO}}_{2}}$; solid circles: HR; open squares: oxy-Hb, deoxy-Hb, total-Hb, and ${\mathrm{StO}}_{2}$ over FPL; solid squares: oxy-Hb, deoxy-Hb, total-Hb, and ${\mathrm{StO}}_{2}$ over FPR. Changes in ${\mathrm{PET}}_{{\mathrm{CO}}_{2}}$ and ${\mathrm{PET}}_{O_{2}}$ were analyzed using repeated-measures ANOVA in conjunction with the Bonferroni *post hoc* test for multiple comparisons. ##, ### indicate significant differences from the baseline at 2 min before the onset of hypoxic air feeding at P<0.05, P<0.0001, respectively. Effects of hypoxia on changes in hemodynamics in the relatively deeper layer over the frontal poles (time) and differences in hemodynamic responses between FPL and FPR (channel; Ch) were analyzed using two-way repeated-measures ANOVA (Ch × time) in conjunction with the Bonferroni *post hoc* test for multiple comparisons. ##, ### indicate significant differences from the baseline at 2 min before the onset of hypoxic air feeding at P<0.05, P<0.0001, respectively. *, ** indicate significant differences between FPL and FPR at P<0.05, P<0.001, respectively. For the oxy-Hb, deoxy-Hb, total-Hb, and ${\mathrm{StO}}_{2}$ data, the asterisks indicate differences between FPL and FPR only. Abbreviations: ${\mathrm{PET}}_{{\mathrm{CO}}_{2}}$, partial pressure of end-tidal carbon dioxide (Torr); ${\mathrm{PET}}_{O_{2}}$, partial pressure of end-tidal oxygen (Torr); HR, heart rate (beats/min); oxy-Hb, oxygenated hemoglobin; deoxy-Hb, deoxygenated hemoglobin; total-Hb, total hemoglobin; ${\mathrm{StO}}_{2}$, tissue oxygen saturation; FPL, left frontal pole; FPR, right frontal pole; SD, standard deviation; ANOVA, analysis of variance.

Figure 2 shows a typical example of changes in the ${\mathrm{PET}}_{{\mathrm{CO}}_{2}}$, ${\mathrm{PET}}_{O_{2}}$, HR, deoxy-Hb, total-Hb, oxy-Hb, and ${\mathrm{StO}}_{2}$ over the forehead in response to isocapnic hypoxia in one of the subjects. The HR increased in response to hypoxia, deoxy-Hb increased, and oxy-Hb decreased. Although total-Hb increased above baseline, ${\mathrm{StO}}_{2}$ decreased. Differences were observed between FPL and FPR in these NIRS parameters except total-Hb. Figure 3 shows the averages of changes in ${\mathrm{PET}}_{{\mathrm{CO}}_{2}}$ and ${\mathrm{PET}}_{O_{2}}$, the HR, hemodynamics, and ${\mathrm{StO}}_{2}$ over the forehead with respect to time after baseline for 15 young adults. From baseline to hypoxia, ${\mathrm{PET}}_{O_{2}}$ decreased from ∼100 to ∼45  Torr, whereas ${\mathrm{PET}}_{{\mathrm{CO}}_{2}}$ remained unchanged. The HR increased from 65±9 to 74±9  beats/min. By visual inspection, it appeared that both deoxy-Hb over FPL and deoxy-Hb over FPR increased by ∼0.4  μM above baseline in response to hypoxia. In contrast, oxy-Hb over FPL and that over FPR decreased by ∼0.2  μM below baseline. Because the changes in deoxy-Hb were greater than those in oxy-Hb, total-Hb over FPL and that over FPR increased by ∼0.2  μM above baseline. Nevertheless, ${\mathrm{StO}}_{2}$ over FPL and that over FPR decreased by ∼6% below baseline. Two-way repeated-measures ANOVA was performed to confirm these results with respect to changes in hemodynamics. Table 1 shows the results. Two-way repeated-measures ANOVA (Ch × time) for changes in deoxy-Hb over FPL and that over FPR in response to hypoxia showed a significant main effect of time and Ch as well as an interaction effect. Similarly, two-way repeated-measures ANOVA (Ch × time) for changes in oxy-Hb showed a significant main effect of time and Ch as well as an interaction effect. Moreover, two-way repeated-measures ANOVA (Ch × time) for changes in ${\mathrm{StO}}_{2}$ showed a significant main effect of time and Ch as well as an interaction effect. Finally, two-way repeated-measures ANOVA (Ch × time) for changes in total-Hb showed a significant main effect of time only. There was no significant main effect of Ch or an interaction effect.

**Table 1:** Results of two-way repeated-measures ANOVA for hemodynamic responses and oxygenation changes in the relatively deeper layer over the forehead in response to hypoxia.

Items	Variables	Results of two-way repeated measures ANOVA			
		df	F	P	$P\eta^{2}$
Deoxy-Hb	Ch	1.00	10.10	0.007**	0.42
	Error	14.00	—	—	—
	Time	2.80	230.52	0.000***	0.94
	Error	39.26	—	—	—
	Ch* time	1.50	6.98	0.008**	0.33
	Error	20.97	—	—	—
Oxy-Hb	Ch	1.00	17.62	0.001**	0.56
	Error	14.00	—	—	—
	Time	3.65	61.14	0.000***	0.81
	Error	51.08	—	—	—
	Ch* time	3.01	8.50	0.000***	0.38
	Error	42.14	—	—	—
Total-Hb	Ch	1.00	1.16	0.299	0.08
	Error	14.00	—	—	—
	Time	3.99	30.75	0.000***	0.69
	Error	55.91	—	—	—
	Ch* time	2.81	1.93	0.144	0.12
	Error	39.30	—	—	—
${\mathrm{StO}}_{2}$	Ch	1.00	17.62	0.001**	0.56
	Error	14.00	—	—	—
	Time	3.65	61.14	0.000***	0.81
	Error	51.08	—	—	—
	Ch* time	3.01	8.50	0.000***	0.38
	Error	42.14	—	—	—

The effects of hypoxia on changes in hemodynamics in the relatively deep layer over the forehead (time) and the difference in hemodynamic responses between FPL and FPR (channel; Ch) were analyzed using two-way repeated-measures ANOVA (Ch × time) in conjunction with the Bonferroni *post hoc* test for multiple comparisons. Asterisks indicate significant main effects of time or Ch or interaction at *P<0.05, **P<0.01, and ***P<0.001.

Oxy-Hb, oxygenated hemoglobin; deoxy-Hb, deoxygenated hemoglobin; total-Hb, total hemoglobin; ${\mathrm{StO}}_{2}$, tissue oxygen saturation; FPL, left frontal pole; FPR, right frontal pole; ANOVA, analysis of variance.

### 3.2 Effect of Isocapnic Hypoxia on HR and HRV

Table 2 shows HRV at baseline and during hypoxia. From baseline to hypoxia, the mean R–R interval decreased (P=0.001), the mean HR increased (P=0.001), and the pRR50 decreased (P=0.023). No other parameters showed statistically significant changes.

**Table 2:** HR and HRV at baseline and during hypoxia.

		Baseline	Hypoxia	p	df	t	ES
RR	Mean	972	849	0.001**	14.00	4.12	0.74
	S.D.	159	133	—	—	—	—
SDRR	Mean	151	144	0.955	14.00	0.26	0.07
	S.D.	191	185	—	—	—	—
HR	MEAN	65	74	0.000***	14.00	−21.97	0.99
	S.D.	9	9	—	—	—	—
RMSSD	Mean	134	123	0.256	14.00	0.25	0.07
	S.D.	208	209	—	—	—	—
pRR50	Mean	0.33	0.27	0.023*	14.00	2.56	0.56
	S.D.	0.19	0.23	—	—	—	—
VLF (${\mathrm{ms}}^{2}$)	Mean	4,534	2,785	0.955	14.00	1.05	0.27
	S.D.	7,751	3,145	—	—	—	—
LF (${\mathrm{ms}}^{2}$)	Mean	8,304	4,745	0.820	14.00	1.04	0.27
	S.D.	15,825	7,663	—	—	—	—
HF (${\mathrm{ms}}^{2}$)	Mean	50,029	39,955	0.532	14.00	0.64	0.17
	S.D.	113,363	100,374	—	—	—	—
VLF (%)	Mean	27	30	0.479	14.00	−0.73	0.27
	S.D.	19	21	—	—	—	—
LF (%)	Mean	26	28	0.659	14.00	−0.45	0.27
	S.D.	12	17	—	—	—	—
HF (%)	Mean	43	38	0.233	14.00	1.25	0.17
	S.D.	23	26	—	—	—	—
LF/HF	Mean	1.03	1.99	0.115	14.00	−1.68	0.41
	S.D.	0.90	2.73	—	—	—	—

Values are presented as mean and SD. Effects of hypoxia on most parameters of HRV were analyzed using Student’s t-test; the exceptions were SDRR and LF/HF, which were analyzed using Wilcoxon’s signed rank test in accordance with the results of normality. Asterisks indicate significant difference between baseline and hypoxia at *P<0.05, **P<0.01, and ***P<0.001, respectively.

Abbreviations: SD, standard deviation; R–R, R–R interval; SDRR, standard deviation of R–R interval; HR, heart rate; HRV, heart rate variability; RMSSD, root mean square of successive differences; pRR50, percentage of adjacent R–R intervals that varied by >50  ms; VLF, very-low-frequency spectral power; LF, low-frequency spectral power; HF, high-frequency spectral power; ${\mathrm{ms}}^{2}$, absolute power; %, relative power; LF/HF, low-frequency to high-frequency ratio.

### 3.3 Correlations of Changes in Total-Hb with HR and HRV

**Fig. 4:**
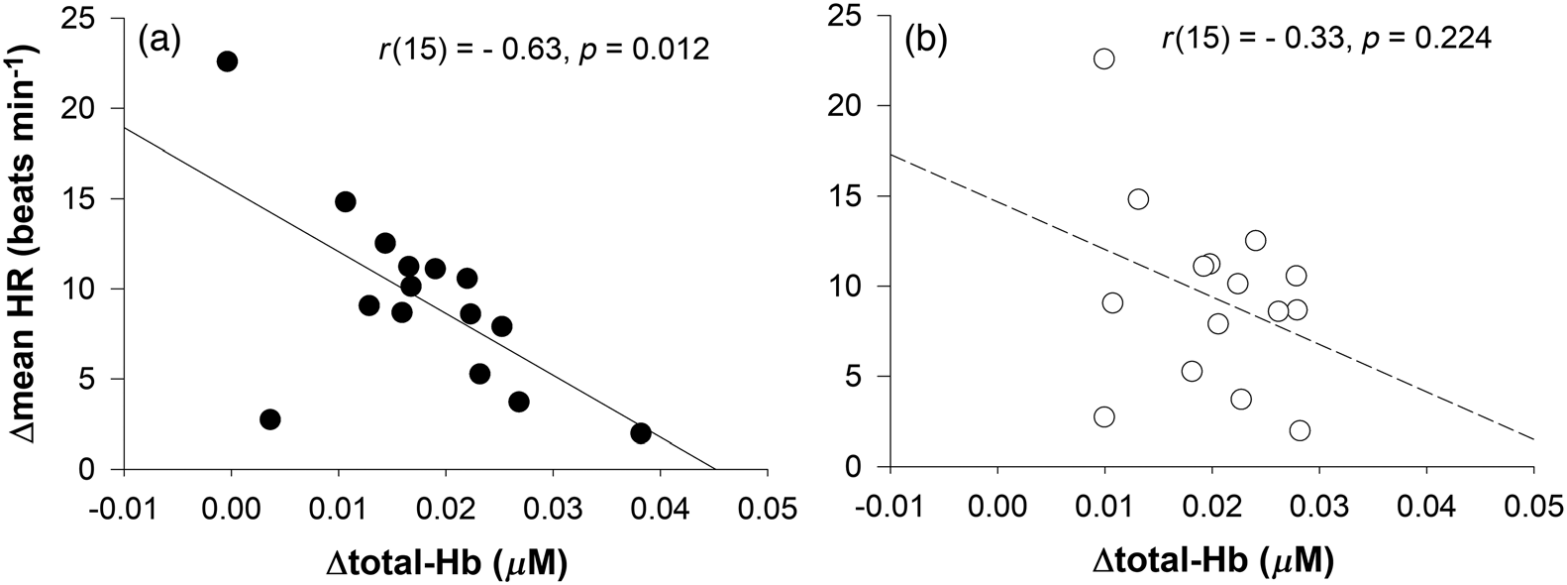
Relationship between changes in total-Hb in the relatively deeper layer over the frontal poles [(a) FPL and (b) FPR] and changes in mean HR. Pearson’s correlation coefficient was used. Abbreviations: total-Hb, total hemoglobin; HR, heart rate; FPL, left frontal pole; FPR, right frontal pole.

Figure 4 shows the relationship between changes in total-Hb over the frontal poles (FPL and FPR) and changes in the mean HR. Although there was a statistically significant negative correlation between changes in total-Hb over FPL and changes in the mean HR (r(15)=−0.63, P=0.012), there was no significant relationship between changes in total-Hb over FPR and changes in the mean HR (r(15)=−0.33, P=0.224). With respect to changes in total-Hb over FPR, there was a trend toward a relationship between total-Hb over FPR and VLF-HRV (r(15)=0.50, P=0.058).

### 3.4 Changes in Hemodynamics in Response to Hypoxia in the Superficial Layer Over the Forehead

**Fig. 5:**
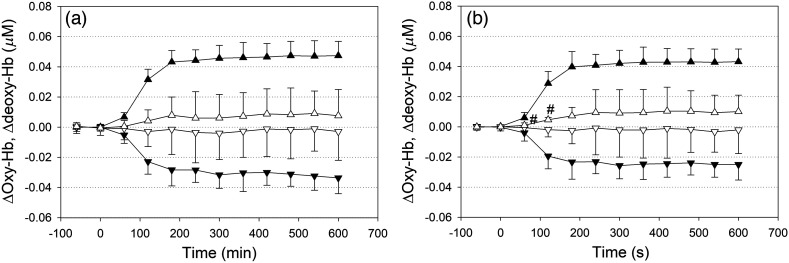
Changes in hemodynamics in the superficial layer over (a) FPL and (b) FPR in response to hypoxia. Changes in hemodynamics in the relatively deeper layer are shown for reference. Each symbol and error bar represents the 60-s mean and SD among n=15 subjects. Solid triangles: oxy-Hb and deoxy-Hb in the relatively deeper layer over FPL and FPR; open triangles: oxy-Hb and deoxy-Hb of the relatively superficial layer over FPL and FPR. Effects of hypoxia on changes in hemodynamics in the superficial layer of the forehead (time) and the difference between FPL and FPR (channel; Ch) were analyzed using two-way repeated-measures ANOVA (Ch × time) in conjunction with the Bonferroni *post hoc* test for multiple comparisons. # indicates significant difference from the baseline at 2 min before the onset of hypoxic air feeding at P<0.05. Abbreviations: FPL, left frontal pole; FPR, right frontal pole; oxy-Hb, oxygenated hemoglobin; deoxy-Hb, deoxygenated hemoglobin; SD, standard deviation; ANOVA, analysis of variance.

Figure 5 shows the changes in deoxy-Hb and oxy-Hb in the superficial layer over FPL and FPR with respect to the time after baseline. For comparison, the changes in deoxy-Hb and oxy-Hb of the relatively deep layer over FPL and FPR are also shown.

Table 3 shows the results of two-way repeated-measures ANOVA for changes in deoxy-Hb and oxy-Hb in the superficial layer over FPL and that over FPR in response to hypoxia. Two-way repeated-measures ANOVA (Ch × time) for changes in deoxy-Hb in the superficial layer over FPL and that over FPR in response to hypoxia showed a significant main effect of time without a main effect of the Ch or an interaction effect. With respect to changes in oxy-Hb in the superficial layer over FPL and those over FPR in response to hypoxia, two-way repeated-measures ANOVA (Ch × time) showed no significant main effects of time or Ch or an interaction effect. Accordingly, although deoxy-Hb in the superficial layer over FPL and that over FPR seemed to increase above baseline to the same extent in response to hypoxia (although most of these changes were not significant), oxy-Hb remained unchanged from baseline. In addition, there was no difference between the changes in deoxy-Hb or oxy-Hb in the superficial layer over FPL and those over FPR.

**Table 3:** Results of two-way repeated-measures ANOVA for changes in deoxy-Hb and oxy-Hb in the superficial layer over the forehead in response to hypoxia.

Items	Variables	Results of two-way repeated measures ANOVA			
		df	F	P	$P\eta^{2}$
Deoxy-Hb	Ch	1.00	0.16	0.695	0.01
	Error	14.00	—	—	—
	Time	1.46	5.13	0.024*	0.27
	Error	20.39	—	—	—
	Ch* time	1.37	0.35	0.632	0.02
	Error	19.22	—	—	—
Oxy-Hb	Ch	1.00	0.01	0.936	0.00
	Error	14.00	—	—	—
	Time	1.70	0.35	0.677	0.02
	Error	23.87	—	—	—
	Ch* time	1.52	0.16	0.791	0.01
	Error	21.29	—	—	—

The effects of hypoxia on changes in hemodynamics in the superficial layer over the forehead (time) and differences between FPL and FPR (Ch) were analyzed using two-way repeated-measures ANOVA (Ch × time). Asterisks indicate significant main effect of time or Ch or interaction at *P<0.05.

Abbreviations: oxy-Hb, oxygenated hemoglobin; deoxy-Hb, deoxygenated hemoglobin; total-Hb, FPL, left frontal pole; FPR, right frontal pole; ANOVA, analysis of variance.

## 4 Discussion

### 4.1 Key Findings in this Study

The two key findings in this study are that asymmetry in prefrontal oxygenation was present in response to hypoxia and that the increase in total-Hb over FPL was inversely associated with the increase in HR.

### 4.2 Prefrontal Asymmetry in NIRS Parameters

Hb $O_{2}$ saturation in arterial blood (${\mathrm{SaO}}_{2}$) as estimated by ${\mathrm{PET}}_{O_{2}}$^30^ decreased from 97.9±0.2% at baseline in normoxia by 17.1±0.9% to 80.8±0.8% during hypoxia, whereas ${\mathrm{StO}}_{2}$ decreased by only 6.4±1.2% over FPL and 5.6±1.4% over FPR in the present study. Such differences between ${\mathrm{SaO}}_{2}$ and ${\mathrm{StO}}_{2}$ imply compensation for Hb $O_{2}$ desaturation by vasodilatation, thus leading to increased cerebral perfusion. Changes in total-Hb can be considered changes in cerebral perfusion,^17^ and total-Hb increased secondary to hypoxia in this study. Other studies using NIRS also demonstrated these hemodynamic responses and oxygenation changes secondary to hypoxia.^16^^,^^22^ This study was conducted to clarify any differences in NIRS parameters between the right and left PFCs in response to hypoxia; there were no differences in total-Hb between the right and left PFCs. Accordingly, there was no prefrontal asymmetry in total-Hb or perfusion. However, in response to hypoxia, there were differences between the right and left PFCs in NIRS parameters, such as oxy-Hb, deoxy-Hb, and ${\mathrm{StO}}_{2}$, thus indicating prefrontal oxygenation asymmetry in response to hypoxia. Because hypoxia affects cerebral vessels over the whole brain, any discrepancies between changes in ${\mathrm{StO}}_{2}$ and changes in total-Hb may be caused by the systemic effects of hypoxia. ${\mathrm{StO}}_{2}$ is primarily influenced by two factors: $O_{2}$ delivery and tissue $O_{2}$ consumption.^31^ Although there were no differences between the left and right PFCs in cerebral perfusion changes, there were differences in ${\mathrm{StO}}_{2}$ changes, implying a difference in $O_{2}$ consumption.

In terms of prefrontal asymmetry, a previous study supports the findings of the current investigation. Papadelis et al.^32^ demonstrated prefrontal asymmetry in EEG signals in response to hypoxia. The authors assessed hypoxia-induced EEG alterations over the PFC and found increases in the power of alpha and theta bands in response to hypoxia. Although there were no hemispheric differences in the power increases of alpha bands over the PFC, power increases of theta bands were observed only over the FPL. The theta wave increases over the PFC occurred without prefrontal asymmetry, similar to what has been reported in subjective sleepiness,^33^ during meditation,^34^ and during recall of haptic information.^35^ The implications of increased theta power over the FPL in response to hypoxia are not clear. Moreover, in a study by Critchley et al.^23^ in which changes in rCBF in response to hypoxia were measured using fMRI, prefrontal asymmetry in the rCBF was observed in response to hypoxia.

With respect to prefrontal oxygenation, it appears that prefrontal oxygenation asymmetry also exists at rest^20^ and that spontaneous oscillations in prefrontal oxygenation occur.^36^ The causes of such prefrontal oxygenation asymmetry remain unknown. However, a study by Ishikawa et al.^36^ showed a positive correlation between the laterality of changes in oxy-Hb with right PFC dominance and the anxiety state at rest. Moreover, a study by Zohdi et al.^20^ showed a positive correlation between the laterality of changes in ${\mathrm{StO}}_{2}$ with right PFC dominance and the pulse-respiration quotient at rest. It appears that the pulse-respiration quotient is associated with the state of the autonomic nervous system.^21^

### 4.3 Changes in HR and HRV in Response to Isocapnic Hypoxia

HR increases in response to hypoxia; this increase is associated with sympathetic activation and/or parasympathetic withdrawal. It has been postulated that sympathetic activation is evoked by peripheral chemoreceptor stimulation,^37^ whereas parasympathetic withdrawal is induced by increased breathing.^38^ With respect to whether HRV might follow these changes in autonomic nerve activity, several studies have reported that certain parameters of parasympathetic nerve activity (RMSSD, pRR50, and HF-HRV) either decrease^18^^,^^39^ or remain unchanged^40^ and that a parameter of sympathetic nerve activity (the LF/HF ratio) either increases^17^ or remains unchanged.^18^ This study demonstrated a decrease in pRR50. Because pRR50 is a parameter of parasympathetic nerve activity, decreased pRR50 likely indicates parasympathetic withdrawal. Although it has been postulated that sympathetic nerve activity increases in response to hypoxia,^37^ studies evaluating the effects of hypoxia on HRV have not always shown increases in the LF/HF ratio;^18^ this study also found no increases. There is thus no apparent study condition in which hypoxia elicits an increase in the LF/HF ratio.

### 4.4 Association Between Hemodynamic Responses to Hypoxia and HR

Hemodynamic responses over the PFC increase in response to cognitive tasks that require a strong mental workload, such as mental arithmetic; these hemodynamic responses are accompanied by increased HR.^1^ In studies by Tanida et al.,^12^^,^^13^ the increase in hemodynamic responses over the right PFC was associated with changes in HR. In this study, however, changes in total-Hb over the left PFC were inversely associated with changes in HR (r(15)=−0.63, P=0.012). These findings may indicate that the left PFC plays a role in attenuating increases in the HR associated with hypoxia. A study by Wittling et al.^41^ suggested that the left cerebral hemisphere is responsible for changes in the HR by affecting parasympathetic nerve activity. The authors found that when the left cerebral hemisphere was activated by a divided visual field paradigm, HF-HRV was enhanced.^41^ Conversely, when the right cerebral hemisphere was activated using a divided visual field paradigm, the stroke volume, cardiac output, and mean systolic ejection rate increased.^42^ Among studies in which the activity of the right or left cerebral hemisphere was selectively attenuated by anesthetic injection into the carotid artery, the implication of the findings did not reach agreement. In a study by Ahern et al.,^43^ the effects of unilateral anesthetic injection into the carotid artery on the HR and HRV were evaluated in patients with epilepsy. Following injection of an anesthetic agent into either the right or left carotid artery, the LF/HF ratio and HR increased. These increases were greater following injection into the right than left carotid artery in all age groups of patients with epilepsy. However, this result was not obtained when the effect of anesthetic injection on the HR was evaluated only in younger patients in a study by Thayer et al.^44^ Instead, they found that the peak HR following anesthetic injection into the left carotid artery was greater than the effect of anesthetic injection into the right carotid artery in patients with epilepsy with a mean age of 20 years.^44^ Similarly, when the left hemisphere was inactivated by anesthetic injection into the left carotid artery in patients with epilepsy with an age of ∼25 years, the LF/HF ratio^45^ and HR^46^ increased, indicating parasympathetic inhibition of the HR by the left hemisphere in young adults.

With respect to changes in total-Hb over FPR, this study showed a trend for a relationship between total-Hb over FPR and VLF-HRV (r(15)=0.50, P=0.058). The physiological implication of VLF-HRV is not yet well defined. Studies using pharmacological blockade have reported that VLF-HRV may be influenced by the renin–angiotensin system^47^^,^^48^ or that it may be associated with parasympathetic nerve activity.^48^ However, VLF-HRV was increased both by shivering associated with a decreased core temperature in a study by Fleisher et al.,^49^ and by physical exercise in a study by Bernardi et al.^50^ These findings suggest that VLF-HRV might be associated with sympathetic nerve activity. Moreover, Shiomi et al.^51^ reported that, in patients with obstructive sleep apnea, VLF-HRV is enhanced during hypoxia associated with respiratory arrest or by respiratory arrest itself. By contrast, Francis et al.^52^ reported that VLF-HRV is decreased by hyperoxia.

### 4.5 Limitations of the Study

With respect to the findings of this study regarding prefrontal oxygenation asymmetry secondary to hypoxia, there may be confounding factors, such as psychological factors. According to the approach–withdrawal model, the left PFC is associated with positive affect and approach-related behavior, whereas the right PFC is associated with negative affect and withdrawal behavior.^53^ Moreover, a study using functional NIRS^54^ reported increased activation in the right PFC when subjects were exposed to negative stimuli, and increased activation in the left PFC when subjects were exposed to positive stimuli. However, it remains uncertain whether prefrontal oxygenation asymmetry in response to hypoxia is applicable to the approach–withdrawal model.

A further limitation of this study is that the subjects did not have any experience with this kind of study; some of them might have felt anxious about being exposed to hypoxic gas or the other experimental procedures. Moreover, with respect to the structural differences between hemispheres, it has been reported that asymmetry may exist in frontal lobe volumes.^55^^,^^56^ However, it remains unknown how such structural asymmetry might affect the results of this study.

Another concern of this study is its low statistical power. A power analysis indicated that the statistical power of the data for changes in total-Hb was sufficiently high (>0.95) to detect differences between FPL and FPR. However, the statistical power of the data for the relationship between the HR and total-Hb was <0.8; the power analysis indicated that ∼10 more samples were required to obtain sufficient power (0.8).

In this study, changes in oxy-Hb and deoxy-Hb over the frontal poles were measured using spatially resolved NIRS. NIRS measures changes in oxy-Hb and deoxy-Hb within illuminated areas, including both intracranial and extracranial tissues. Changes in oxy-Hb and deoxy-Hb, as determined by NIRS, might be affected by changes in scalp blood flow. It appears that changes in oxy-Hb and deoxy-Hb as determined by NIRS over the forehead would be contaminated by an increase in skin blood flow during exercise. Because hypoxia also enhances skin blood flow,^24^ it may be argued that such an increase in skin blood flow during hypoxia affected the hemodynamic responses and oxygenation changes determined by NIRS in this study.^57^ Figure 5 shows the changes in oxy-Hb and deoxy-Hb in the superficial layer of the head together with those of the relatively deeper layer. Two-way ANOVA (Ch × time) for changes in deoxy-Hb in the superficial layer over FPL and that over FPR in response to hypoxia showed a significant main effect of time without a main effect of Ch or an interaction effect. However, at most time points, changes in deoxy-Hb in the superficial layer were insignificant with respect to the baseline. With respect to changes in oxy-Hb in the superficial layer over FPL and those over FPR in response to hypoxia, two-way repeated-measures ANOVA (Ch × time) showed no significant main effects of time or Ch or an interaction effect. Compared with those from the relatively deeper layer of the forehead, the NIRS signals from the superficial layer of the forehead were apparently smaller. Moreover, there was no right- and left-side differences in the NIRS signals from the superficial layer of the head.

Changes in blood pressure might affect both CBF and hemodynamic responses to hypoxia. CBF increases in response to hypercapnia, and this increase is affected by changes in blood pressure.^58^ The hemodynamic responses observed in this study might thus have been affected by changes in blood pressure. However, because blood pressure measurements were unsuccessful in this study, it remains unknown how the results were affected by blood pressure. Nevertheless, it is unlikely that hemodynamic responses over FPL and FPR are affected differently by changes in blood pressure.

### 4.6 Summary and Perspectives

This study was conducted as part of a project to investigate the relationships among CVR, cognitive function, and aerobic capacity in older adults. The main questions of the project were whether regular physical activity might lead to better cerebrovascular function, and whether better cerebrovascular function might lead to better cognitive function in older adults, whose cerebrovascular and cognitive health is postulated to be attenuated with aging. Before proceeding with the study in older adults, it was first necessary to clarify whether a difference exists between the right and left PFCs in NIRS-determined CVR in response to hypoxia in young adults, whose cerebrovascular and cognitive health is normal (as a reference). It was also necessary to clarify whether CVR in response to hypoxia is associated with HR responses. This will help to elucidate whether changes in hemodynamic responses and oxygenation changes secondary to hypoxia may simply reflect the direct effects of hypoxia on cerebral vessels or whether other physiological mechanisms may be involved. By conducting isocapnic hypoxia tests in young men, this study revealed no differences in NIRS-determined hemodynamic responses (i.e., total-Hb) to hypoxia between the left and right PFC, thus indicating no differences in CVR between the left and right PFC. However, the observed asymmetry in ${\mathrm{StO}}_{2}$ with FPL dominance, as well as the correlation between changes in HR and changes in total-Hb over the FPL, indicate possible differences between FPL and FPR in the mechanisms responsible for CVR. One such mechanism may be the CAN, a network of brain regions that regulates and controls autonomic functions.^15^ Although its primary role is to regulate autonomic functions, such as HR, growing evidence suggests that it also modulates cognitive processes.^59^^–^^62^ Further studies are needed to clarify whether NIRS-determined hemodynamic responses and oxygenation changes secondary to hypoxia are associated with the CAN, as well as to investige the relationships among CVR, cognitive function, and aerobic capacity in older adults. Moreover, it will be important to clarify whether NIRS-determined prefrontal oxygenation asymmetry secondary to hypoxia also occurs in older adults or whether hemispheric asymmetry reduction may occur in older adults^63^ in prefrontal oxygenation secondary to hypoxia.

## 5 Conclusion

NIRS-determined hemodynamic responses and oxygenation changes secondary to hypoxia might not simply reflect the direct effect of hypoxia on cerebral vessels. This study showed that prefrontal oxygenation asymmetry was present in response to hypoxia whereas there was no prefrontal asymmetry with respect to the change in total-Hb. Moreover, the change in total-Hb over FPL was associated with the change in HR.
